# Determinants of content marketing effectiveness: Conceptual framework and empirical findings from a managerial perspective

**DOI:** 10.1371/journal.pone.0249457

**Published:** 2021-04-01

**Authors:** Clemens Koob

**Affiliations:** Department of Health and Nursing, Katholische Stiftungshochschule München, Munich, Germany; West Pomeranian University of Technology, POLAND

## Abstract

Content marketing has gained momentum around the world and is steadily gaining importance in the marketing mix of organizations. Nevertheless, it has received comparatively little attention from the scientific community. In particular, there is very little knowledge about the effectiveness, optimal design and implementation of content marketing. In this study, the authors conceptualize content marketing as a set of activities that are embedded in and contingent on the specific organizational context. Based on this framework, the authors empirically investigate the context features determining content marketing effectiveness from a managerial perspective, using primary data collected from senior marketers in 263 organizations from various sectors and across different size categories, conducting multiple regression analysis. The empirical results indicate that clarity and commitment regarding content marketing strategy and a content production in line with the organization’s target groups’ content needs as well as normative journalistic quality criteria are context factors associated with higher content marketing effectiveness. The outcomes also reveal that regularly measuring content marketing performance and using the data obtained as guidance for improving content offerings positively influence content marketing effectiveness, as do structural specialization and specialization-enabling processes and systems. The insights provided in this study could offer important theoretical contributions for research on content marketing and its effectiveness and may help practitioners to optimize the design and implementation of content marketing initiatives.

## Introduction

In times when consumers are becoming increasingly skeptical of traditional advertising, organizations need, more than ever, effective alternatives to traditional marketing communications. In these circumstances, content marketing (CM) has gained momentum around the world and is steadily gaining importance in the marketing mix of organizations, complementing traditional marketing instruments [e.g., 1]. CM investments have increased substantially. In the German-speaking area, for example, investments have risen from € 4.4b in 2010 to € 9.4b in 2019 and are forecast to grow further to € 12.5b by 2023 [2].

Content marketing refers to the creation and distribution of relevant, valuable brand-related content to current or prospective customers or other target groups (e.g. jobseekers, employees or investors) via digital platforms or print media to drive strategic business objectives [3–5]. Unlike traditional advertising, which typically denotes a form of communication designed to persuade or even push target groups to take some action, now or in the future [6], content marketing focuses on adding value to their lives, for instance by educating them, helping them solve problems, entertaining them or supporting them make well-informed decisions. Thus, content marketing is based on the social exchange theoretical principle that an organization’s delivery of valuable content to a target group will see it rewarding the organization in exchange with positive attitudes (e.g. brand trust) or behaviors (e.g. brand related interactions).

However, despite content marketing’s growing importance, it has received comparatively little attention from the scientific community [3, 5]. So far, research has primarily focused on definitions and conceptualizations of content marketing [e.g. 3, 5, 7, 8] and potential consumer- and firm-based consequences. Besides, there is a limited number of exploratory analyses and investigations about the effectiveness of content marketing that focus on specific sectors and types of media. Wang et al. [4], e.g., found CM effectiveness in the B2B domain to depend on the frequency of customers’ content consumption. Taiminen and Ranaweera [9] identified specific helpful brand actions, i.e. approaching content marketing with a problem-solving orientation, as increasing the effectiveness of B2B content marketing. With respect to consumers and branded social content, Ashley and Tuten [10] identified frequent updates, incentives for participation, as well as experiential, image and exclusivity messages to be associated with effectiveness. Chwialkowska’s study [11] revealed that customer-centric as opposed to brand-centric social content is more effective. Also, Liu and colleagues [12] provided evidence that short video clips can be effective to drive usage of other branded online content. However, apart from such rather focused studies, we have very little overall knowledge about the effectiveness of content marketing. In particular, and as Hollebeek and Macky [3] noted, still “little is known regarding its optimal design and implementation”. The question “what are the key factors for effectiveness” has long been an important theme in the marketing communications literature, but academic understanding regarding the determinants of content marketing effectiveness lags behind to date [3], generating an important knowledge gap that we address in this paper.

To investigate this gap, we conceptualize content marketing from an activity-based perspective. In line with the activity-based perspective of marketing [13, 14], we propose to view content marketing as a set of specific activities, comprising content marketing strategizing, content production, content distribution, content promotion, performance measurement and content marketing organization. Referring to the concept of embeddedness [15, 16], we further assume that these content marketing activities are rooted in and contingent on the specific organizational context, and that particular context features are potential determinants of content marketing effectiveness. Based on this framework, we will empirically investigate the features driving content marketing effectiveness.

Our contribution is as follows: As far as we know, the determinants of content marketing effectiveness have not yet been empirically investigated from a broader perspective. We therefore first provide a theoretical framework for analyzing content marketing effectiveness. Second, we offer empirical insights that could help marketers to potentially improve the design and implementation of their content marketing initiatives, which researchers have called for [3, 5]. Third and in doing so, we might help to move the research on content marketing effectiveness beyond the prevailing anecdotal to an evidence-based level. Fourth, for scholars, this research could offer a platform for further studies into the drivers of content marketing effectiveness. Taken together, these advances could extend current academic and managerial discussions of how to achieve effective marketing communications.

## Theoretical framework and derivation of hypotheses

Any empirical investigation of the determinants of content marketing effectiveness requires a proper conceptualization of CM effectiveness. Hence, the next section proposes such a conceptualization. After that, we propose that content marketing activities take place in an organizational context [15, 16] affecting their effectiveness. Context refers to the specific intra-organizational circumstances, environments and constellations of forces shaping the character of the content marketing activities and their outcome [17]. We outline the potentially relevant context dimensions, being content marketing strategizing, content production, content distribution, content promotion, content marketing performance measurement, and content marketing organization, respectively.

### Content marketing effectiveness

Based on a literature review ([3–5, 8, 18–23], see S1 Appendix for details), content marketing activities can be seen as effective if they trigger superior levels of cognitive, emotional and behavioral customer engagement at the appropriate points throughout the customer journey, strengthen customers’ brand trust and induce favorable brand attitudes, and increase customers’ perceived value of a brand, leading to more favorable responses to the brand and its communications, and thus helping the focal organization reach its strategic business objectives.

### CM effectiveness and CM strategizing

Porter and McLaughlin [15] conclude that there is no universally agreed-upon set of components that comprise the relevant organizational context dimensions. However, they point to the strategizing context to be one of them, i.e. the constellations under which strategizing in the sense of ‘doing of strategy’ unfolds [15]. Strategy research supports the idea that strategic clarity is one aspect of the strategizing context that plays a key role regarding effectiveness since it gives direction and provides orientation [24, 25]. This is also in line with goal setting theory which posits that specific and well-defined challenging goals lead to higher performance [26]. Strategy research also suggests strategy commitment, which can be defined as the extent to which managers and employees comprehend and support the goals and objectives of a strategy [27, 28], as an essential aspect, as it is known to affect strategy supportive behavior. We assume these two factors to be pivotal for content marketing effectiveness, too. In the content marketing domain, strategizing comprises, e.g., the crafting of a content marketing mission and vision, the definition of objectives, the identification and prioritization of target groups, the specification of the unique value an organization is looking to provide through its content, the clarification of key stories to be communicated, or decisions regarding the platforms that will be used to disseminate content [e.g., 5]. A clearly defined content marketing strategy that is communicated and understood within the organization might positively influence CM effectiveness, because it allows to select those CM projects which promise a high strategy contribution. In case commitment to a content marketing strategy is high, all managers and employees might show vigor, get engaged and take personal responsibility for the successful realization of the content marketing initiative. Thus, we expect:

***Hypothesis 1*: *Content marketing is more effective when organizations have a stronger CM strategizing context characterized by strategic clarity and commitment***.

### CM effectiveness and content production

Furthermore, we suggest a strong content production context will be positively related to CM effectiveness. By this, we refer to content production environments in which high quality content can be created [5]. The necessity to create and provide quality content is widely acknowledged in the CM literature [e.g. 5], as it is assumed that quality content is more likely to be interacted with. However, this raises the question of what constitutes quality content. Uses-and-gratifications-theory supports the idea that people seek out media that satisfy their needs and lead to gratification [29, 30]. From this perspective, consumers may select content for functional (e.g. learning about brands, self-education), hedonic (e.g. entertainment, diversion, relaxation) or authenticity motives (e.g. identity construction, self-assurance) [3, 30]. In addition to that, research proposes that ‘quality content’ not only has to meet consumers’ subjective standards, but also certain objective specifications or normative principles. The criteria mentioned in the literature typically include aspects like timeliness, objectivity, accuracy, or diversity of viewpoints [31–36]. Hence, we believe:

***Hypothesis 2*: *A strong content production context*, *characterized by efforts to optimize customer-perceived content value and to adhere to normative quality criteria should be associated with higher content marketing effectiveness***.

### CM effectiveness and content distribution

We assume a specific content distribution context will also be positively related to CM effectiveness. The content distribution context refers to the conditions under which content is distributed and particularly includes the media platforms (e.g. customer magazines, digital magazines, blogs, podcasts, social media, chatbots etc.) used [3, 5, 8]. Research generally supports the idea that communications efforts using multiple media platforms are more effective than initiatives using only a single medium [e.g. 37, 38]. According to Voorveld et al. [39], two psychological processes play a role in explaining these effects. First, forward encoding implies that the exposure to content in the first medium primes interest in the content in the second medium, which in turn stimulates deeper processing and easier encoding of the second content piece, resulting in multiple content retrieval cues and higher effectiveness. Second, multiple source perception refers to the effect that consumers perceive cross-media communications as more expensive, leading to the belief that the communicating brand has to be popular and successful, also resulting in more positive communications results. Furthermore, benefits from combining multiple media distribution platforms might arise from accompanying prospects and customers with the appropriate content platforms at the different points in their consideration and buying processes [40]. On the other hand, it could be argued that investment in too many media distribution properties might attenuate the power of communications, because it prevents an organization from focusing its resources on the most suitable platforms [38]. Reactance theory also suggests that communication across multiple media platforms could unfold negative consequences as customers might associate a brands omnipresence at various platforms with increasing pressure from the firm’s communications attempts which could be perceived as obtrusive [41]. Based on these considerations we believe:

***Hypothesis 3a*: *Content marketing is more effective*, *when the content distribution context is characterized by the usage of an intermediate number of media platforms***.

Content marketers continue to watch out for new opportunities to reach customers and, over time, have shifted content distribution budgets away from print media such as customer magazines to digital media such as digital magazines, blogs, social media and the like [2]. The question is whether and to what extent this shift is beneficial for improving CM effectiveness. Communications theory implies that for effective communication, the sender should match the channel that the receiver prefers [42]. Based on this recommended practice of media matching, organizations ought to be cognizant of customers’ media platform preferences as well as actual media use and adjust their channel choices accordingly. With regard to media preferences, research has repeatedly revealed a high level of consumer conservatism, indicating that established media channels, especially print media, retain favored attributes such as trust, high perceived value, intimacy or visual power, whereas digital media are, e.g., more strongly associated with speed, convenience and efficiency [42, 43]. Considering media use, two models predict different relationships between new and established media. The displacement model assumes increases in new media use will go along with declines in the use of established media (e.g. due to functional advantages of new media or limited time budgets [44, 45]). The complementary model hypothesizes new media usage has no or even a positive effect on established media use within a content domain, as people “interested in procuring information in a particular content area expose themselves to a multitude of media outlets to optimize the information on that particular content area” [46]. Recent studies [45, 47] have provided evidence that adoption of new platforms is reducing the consumption of established media, but that established media will not be fully displaced. Other theoretical accounts also suggest not to neglect print media for digital media. Psychological ownership theory implies that print media, being physical goods, might have a greater capacity to garner an association with the self than digital media, leading to greater value ascribed to them [48]. Regarding text-based content, educational research points to the fact that reading on paper leads to significantly better content comprehension than reading digitally [49], possibly due to better spatial mental representation of the content and more visual and tactile cues fostering immediate overview of the content. Consequently, we expect:

***Hypothesis 3b*: *Content marketing is more effective*, *when the content distribution context is characterized by a joint deployment of print and digital media platforms***.

### CM effectiveness and content promotion

Furthermore, we propose the content promotion context is key for CM effectiveness. Content promotion refers to any paid measures an organization takes to draw attention to its content or to stimulate interest in or usage of its content, typically with the help of or on third-party platforms, with the aim of optimizing content reach. Instruments include, amongst others, influencer marketing, social media and search engine advertising, or classic public relations [50]. Research has repeatedly suggested an attention economy [e.g., 51], denoting a world where people are awash in content, and where peoples’ available time and attention spans are limited, creating an environment in which content competes for customers’ time and attention as scarce resources. Under these circumstances, we expect that paid content promotion measures can help to accentuate content and draw attention to potentially relevant and valuable content pieces, so that these pieces can break though the “content clutter” [52].

Furthermore, the power law of practice and cognitive lock-in theory [43, 53] state, that when people practice specific tasks, the repetition of these tasks increases efficiency, which induces familiarity, from which in turn people are inclined to get cognitively locked-in to the respective media environment. Cognitive lock-in thus denotes a condition wherein a consumer has learned how to use a specific media environment, thanks to multiple interactions with it, with the effect that more familiarity decreases his propensity to search for and switch to competing media alternatives. Research has demonstrated these effects for websites [53, 54], as well as for print media [43]. We believe this thinking may be applicable for a broad range of media environments and applying it to the content marketing context leads us to believe that if customers are already accustomed to use specific content offerings, they see no need to switch to a new content offering. Under these conditions, paid content promotion measures might help to stimulate customers to try a focal organization’s content offer, potentially breaking up existing and initiating new cognitive lock-in processes, thereby supporting the organization’s attempt to transition customers to its own content offerings. Hence:

***Hypothesis 4*: *Content marketing is more effective when organizations have a stronger content promotion context characterized by comprehensive paid content promotion measures***.

### CM effectiveness and CM performance measurement

We also propose that a strong content marketing performance measurement context within an organization will be positively related to CM effectiveness. Content marketing performance measurement (CMPM) can be defined as establishing metrics related to the organization’s content marketing objectives and measuring and evaluating performance relative to these objectives, for the purpose of providing evidence for effectiveness and efficiency of content marketing activities and optimizing these activities. Previous studies have shown positive performance implications of marketing performance measurement in contexts other than content marketing [e.g. 55–57]. We believe for four reasons, that this also applies to the content marketing domain. First, the attention-based view of the firm accentuates that one of the key characteristics of measurement systems is their property to focus and direct attention of organizational members to important issues [58]. By directing minds at what needs to be done, chances increase that it will get done. Thus, we expect, that content marketing performance measurement will get an organization to attend to essential content marketing objectives and activities. We believe that the presence of CMPM activates managers and employees and causes them to achieve coordinated action and to orient their efforts to succeeding on the measured content marketing aspects. Second, previous research [59] has shown that producing measurements is not enough to get the organization into acting, but that organizations are also sensitive to what issues are internally discussed. We argue that CMPM sparks discussions about important content marketing issues, which helps to summon attention and resources for acting, ultimately improving content marketing effectiveness. Third, performance measurement usually allows to monitor the performance of marketing activities, be it relative to prior objectives, similar activities in the past, or other benchmarks, lowering uncertainty about the performance of decisions and about whether the decisions were the right ones, which in turn helps to learn and plan marketing activities producing desired outcomes [56]. We thus expect that CMPM will nurture learning, which in turn will improve content marketing decisions, and thus content marketing effectiveness. Fourth, performance measurement usually includes performance feedback, and previous studies have consistently shown that performance feedback is positively associated with work engagement [60]. Higher work engagement in turn implies that managers and employees invest more energy into their work roles, leading to superior work outcomes [61]. Thus, we expect that CMPM energizes organizational actors to act in desired ways to meet the organization’s goals. Hence:

***Hypothesis 5*: *Content marketing is more effective when organizations have a stronger content marketing performance measurement context***.

### CM effectiveness and content marketing organization

Finally, we expect a strong content marketing organization will be positively related to CM effectiveness. Porter and McLaughlin [15] indicate that organizational structures and processes are one of the major components contextualizing activities within an organization. Research on marketing organization also highlights the importance of organizational structures and processes for marketing performance [62, 63]. It is widely acknowledged in the marketing literature, that organizations face dynamic and complex marketing communications environments, e.g. in terms of the development and transformation of technology and media or consumer behavior evolving at an increasingly rapid pace [6]. Under these conditions, specialization and autonomy seem to be favorable characteristics of organizational structure [64]. Specialization denotes the level to which activities in the organization are differentiated into unique elements, while autonomy refers to the level to which employees have control in executing those activities. Organizations high in specialization and autonomy have a high share of specialist employees who direct their efforts to a clearly defined set of activities, and as experts with specialized knowledge in their particular work areas, they enjoy substantial autonomy to determine the best approach to carry-out their tasks [65]. According to prior research, the combination of specialization and autonomy enables an organization to assign tasks to those employees who are best able to perform them, it enhances the organization’s knowledge base, and it promotes the development of innovative ideas and solutions [62, 63, 66]. However, research has also indicated that specialized organizational structures with high degrees of autonomy need the support of adequate processes and systems to function properly [62].

The application of this thinking to content marketing leads us to two considerations: First, we believe that, also in this domain, structural specialization coupled with autonomy could be beneficial. It could allow an organization to assign content marketing tasks to managers and employees that are best prepared to tackle them. Further, specialization could enhance an organization’s content marketing knowledge base, foster the development of innovative content marketing ideas and solutions and enable the organization to quickly respond to upcoming communication needs. An example for such a structure could be a dedicated content marketing unit with a high share of task- and skill-specialized content marketing experts that have control over how they organize their work and that have significant autonomy in making decisions. Second, we assume that an increase in content marketing specialization and autonomy within an organization also demands processes and information technology systems with a proper fit [67]. We believe that processes and systems are required that enable and support interaction and collaboration between content marketing specialists, between content marketing experts and further marketing functions, and also between content marketing experts and other relevant organizational entities. To sum up, we posit:

***Hypothesis 6*: *Content marketing is more effective when organizations have a stronger content marketing organization***.

**Fig 1:**
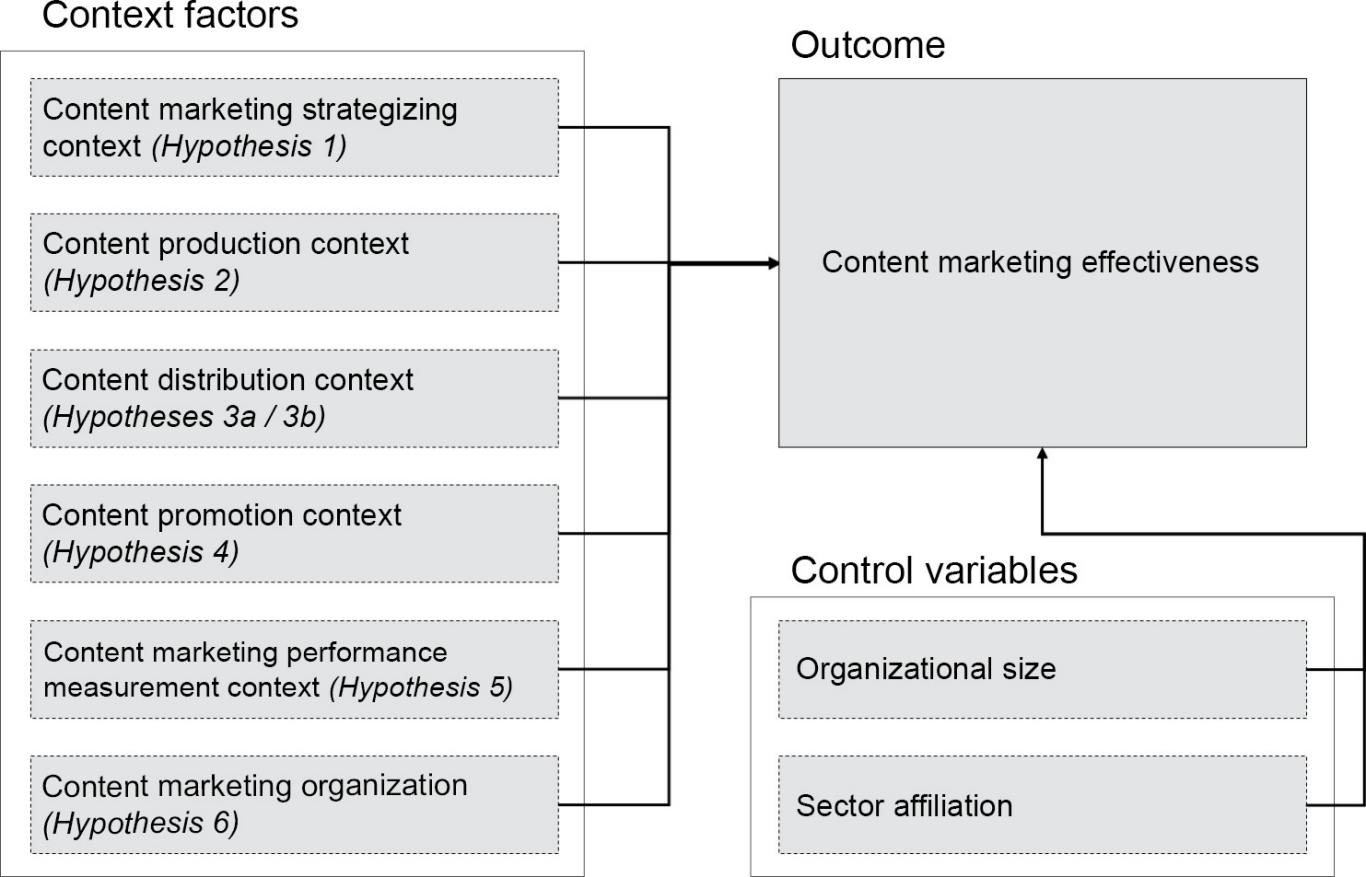
A model of the determinants of content marketing effectiveness.

Fig 1 provides a summary of the proposed theoretical framework.

## Method

### Data collection and sample

We gathered data from organizations with over 250 employees in the German-speaking area, that is Germany, Switzerland, and Austria. Regarding industry characteristics, organizations from all sectors in line with the business registers of the three countries, comprising a broad range of industrial, services, finance and trade sectors, were eligible to take part in the investigation. We targeted medium- and large-sized organizations because they are more likely to employ complex marketing practices such as content marketing. All data were collected using an online survey with the sample drawn from an online panel provider. There is profound evidence from prior research that online panel data is capable of delivering high-quality data outcomes [68]. Porter et al. [68] recommend using online panel data particularly for studies requiring access to specific populations. Referring to this guidance, online panel data and the online panel provider Norstat were deliberately chosen for this study, because it required access to the very specific population of senior marketing or communications directors, and people in equivalent positions, responsible for the respective firms’ content marketing activities, as key informants, with the online panel provider being capable of recruiting this hard-to-reach sample. The aforementioned group of managers was identified as key informants because they are organizational members who can provide reliable data on the organizations’ content marketing activities and effectiveness. Data collection was carried out in accordance with further recommendations compiled from the literature by Porter et al. [68] regarding participant recruitment, selection and information and data quality measures. We captured participants’ managerial positions and involvement in content marketing activities in a screener survey to verify key informant appropriateness and reduce potential key informant bias, used attention checks and applied lower and upper limits of survey completion time to ensure high-quality responses, and captured IP addresses to control for potential multiple responses from the same managers.

Before carrying out the study, the University Ethics Review Board regulations indicated that a research ethics review was not required. Reasons for this decision are that the investigation does not include any manipulations or vulnerable groups, and participants were guaranteed that their data is treated anonymously. Moreover, the data has been collected consistent with the ethical guidelines of the Academy of Marketing Science and in accordance with the EU General Data Protection Regulation. All participants provided informed consent by clicking on the link to start the study, participation was completely voluntary, and only data from participants were used who fully completed the study.

In total, data collection yielded 319 responses. The sample comprised 53 managers from organizations that do not apply content marketing practices and 3 executives that failed to pass the aforementioned data quality checks. We therefore eliminated those respondents from the sample. Hence, the final sample comprised the answers from 263 organizations.

The characteristics of respondents were in line with our expectations of key informants. We were successful in getting senior-level marketing and communications executives as respondents: 131 were board members such as CMOs, 56 were marketing vice presidents or directors, 38 were corporate communications vice presidents or directors, 36 were vice presidents or directors of a dedicated content marketing unit, and the remaining 2 were senior executives in other marketing communications functions. Of the 263 organizations in our sample, 125 were from the services sector, 67 from the industrial sector, 51 from the finance sector, and 20 from the trade sector. Regarding size, 69 organizations had between 250 and 499 employees, 58 had 500 to 999 employees, 72 had between 1,000 and 4,999 employees, and 64 employed a workforce of 5,000 or more people.

### Measures

For collecting data, we relied on a structured questionnaire. Whenever possible, we used measures from previous research and modified them for our study. All questions were asked in German language. The measures of the main variables are displayed in the table in S1 Table.

#### Dependent variable

*Content marketing effectiveness (CMEFFECT)*. To capture the degree of achieved content marketing effectiveness, we asked senior marketing and communications executives for their evaluations. For assessing attained customer engagement as aspect of content marketing effectiveness, we adapted three items from the consumer brand engagement scale which was developed by Hollebeek et al. [69]. These questions capture the managerial assessment of the extent to which focal content marketing activities foster positive brand-related cognitive, affective and conative activity, i.e. consumers’ brand processing, affection, and activation. To assess content marketing’s effects on brand attitudes and perceived brand value as further aspects of content marketing effectiveness, we adapted four perceptual items drawn from Sirdeshmukh et al. [70] and Sengupta and Johar [71]. These questions capture the managerial assessment of the degree to which the respective organization’s content marketing activities trigger brand trust in terms of credibility (expectancy that a promise made by the brand can be relied upon) and benevolence (confidence in the brand motives) and contribute to favorable brand evaluations. Responses to all items of content marketing effectiveness were given on 5-point agreement scales (1 = strongly disagree and 5 = strongly agree). An exploratory factor analysis delivered a one-factor solution; thus, we averaged all items to calculate the overall index of content marketing effectiveness. Cronbach’s alpha coefficient for content marketing effectiveness was .88, exceeding the recommended minimum of .70, indicating a very good reliability [72].

#### Independent variables

*Content marketing strategizing context (CMSTRAT)*. The content marketing strategizing context was assessed using a four-item scale that measured whether the organization had a defined, comprehensible, long-term content marketing strategy and to what extent managers and employees support the strategic direction. The items for strategic clarity and strategy commitment were adapted from related scales developed by Bates et al. [73] and Noble and Mokwa [74]. Responses were given on 5-point agreement scales (1 = strongly disagree and 5 = strongly agree).

*Content production context (CPROD)*. We assessed the content production context using a three-item scale. The items rest on previous research by Hollebeek and Macky [3], Urban and Schweiger [35] and Chen and colleagues [75] and include an organization’s efforts to optimize customer-perceived content value, to adhere to normative content quality criteria, and to plan and create content systematically. Responses were given on 5-point agreement scales (1 = strongly disagree and 5 = strongly agree).

*Content distribution context / intermediate number of media platforms (CDIST1)*. In line with previous research by Kabadayi and colleagues [76], we used a single item to measure the number of media platforms the organizations used for content distribution purposes. We presented our respondents with the following seven media platform alternatives and asked them to mark the ones used by their organizations: customer magazines or newspapers, corporate books, company reports, owned digital media (websites, apps, newsletters, blogs), organic social media, paid social media and emerging platforms (e.g. chatbots, voice assistants). We developed this list on the basis of a review of the academic and trade literature combined with prestudy interviews of content marketing executives. Although we intended the list to be comprehensive, we asked respondents with media platforms not included in the list to add those platforms in a space that was provided. The measure of platform number was simply the number of platforms that each organization used. The range on this item was 1 to 7 platforms. Based on this item, we calculated our measure so that the usage of the intermediate number of four media platforms was assigned the maximum value 4, while lower or higher number of platforms used were assigned values in the range between 1 and 3.

*Content distribution context / joint deployment of print and digital media platforms (CDIST2)*. To operationalize the joint deployment of print and digital media platforms in content distribution, we asked respondents–as done in prior research [77]–how much of their content distribution budgets their organizations were allocating to print or digital media platforms, respectively, with the percentages summing up to 100 percent. We used this information to construct the joint deployment score for each organization and assigned values between zero (print or digital only) and fifty (balanced budget shares) to reflect joint platform usage.

*Content promotion context (CPROM)*. To measure the weight organizations attached to content promotion, respondents were requested to state the share of overall content marketing investments that their organizations allocated to content promotion measures. We adapted this approach from Fam and Yang [77] because marketing executives are usually sensitive to budget information, hence they would feel more comfortable in providing the relative weight of content promotion budgets rather than an absolute figure, leading to more accurate data. The range on this item was 0 to 100 percent.

*Content marketing performance measurement context (CMPERME)*. We assessed the CM performance measurement context using a three-item scale. The items rest on previous research by O’Sullivan and colleagues [55] and Mintz and Currim [56]. They capture content marketing performance measurement frequency regarding deployed print and digital content platforms as well as actual performance measurement data use in terms of the employment of data as guidance for continuously improving content offerings. Responses were given on 5-point agreement scales (1 = strongly disagree and 5 = strongly agree).

*Content marketing organization (CMORG)*. To capture structural specialization and autonomy in the content marketing domain and specialization-enabling processes and systems, we used four questions based on prior research by Olson et al. [63], Walker and Ruekert [66], Barclay [78] and Škrinjar and Trkman [79]. These questions capture the presence of dedicated content marketing units, task- and skill-specialized, autonomous content marketing experts, and processes and information technology systems that enable collaboration of specialized staff and units. Responses were given on 5-point agreement scales (1 = strongly disagree and 5 = strongly agree).

#### Control variables

In addition to the above variables, we considered control variables in our analyses. We followed recommendations for control variable use in the literature that suggest a focused use of controls to not unnecessarily loose available degrees of freedom and statistical power [80, 81]. We also opted for a focused approach to avoid increase in questionnaire length, because this commonly leads to higher response burden [82], which is associated with lower response rates and more response biases. First, we included *organizational size (SIZE)* as a control variable. Size is established to potentially confound marketing practices [83] and organizational performance measures [84]. For example, compared to larger organizations, smaller organizations were found to be more informal with regard to marketing planning and to use fewer ways to measure performance [83]. Thus, organizational size may relate to an organization’s content marketing activities and CM effectiveness. Organizational size was measured by asking the key informants for the number of full-time employees, referring to four size categories. Three dummy variables were used, concerning organizations with 500 to 999, 1,000 to 4,999, and 5,000+ employees, respectively. Organizations with 250–499 employees served as the comparative category. Second, we also controlled for an organization’s *sector affiliation (SECTOR)*. A dummy-coded variable (0 = industrial sector and 1 = services sectors) was assigned to the participating organizations. The rational for selecting sector affiliation as control was that it is well established that sector characteristics, in particular differences between industry and services, play an important role for organizational behavior and outcomes [85]. Examples for sector-specific features are legal restrictions, competitive specifics, ethical concerns, or customer specifics [86]. In content marketing it could, e.g., be that creating attractive, compelling content is harder for organizations in industrial sectors.

### Measure validation and analytical approach

#### Measure validation

As our data met sample size recommendations [87], we assessed the ***validity of*** our ***measures*** using confirmatory factor analysis. The analysis was performed using the lavaan package in R. We estimated a measurement model with the seven reflective constructs in our study (CMSTRAT, CPROD, CDIST1, CDIST2, CPROM, CMPERME and CMORG). Regarding the inclusion of the three single-indicator latent variables (CDIST1, CDIST2, CPROM) in the analysis, we followed the recommendations in the literature [88, 89] to fix loadings at “.95 * variance” and to calculate error variance as “sample variance of the indicator * (1 - .85)”, thus separating the single indicators from the latent variables. We used the robust Satorra-Bentler MLM estimator, since the multivariate normality assumption was not met (Mardia Statistics: skew = 41.95, p < .01 and kurtosis = 374.90, p < .01). The results indicate adequate levels of fit (CFI = 0.97, SRMR = 0.04, RMSEA = 0.05, χ^2^/df = 145.5/101), in accordance with the guidelines provided by Hu and Bentler [90].

We assessed ***convergent validity*** of the measures by examining factor loadings. The analysis indicated that all factor loadings are high (ranging from 0.58 to 0.92), in line with the guidelines of Hair et al. [91], and significant. Cronbach’s alphas of all of the measures range from 0.71 to 0.86, surpassing the acceptable level of 0.70, and composite reliabilities also surpass the acceptable level of 0.60 suggested by Fornell and Larcker [92]. Average variance extracted (AVE), reflecting the amount of variance in the indicators that is accounted for by the latent construct, is a more conservative estimate of the validity of a measurement model [92], and was also calculated for each construct. With the exception of CPROD (0.45), the AVE for each construct is greater than the 0.50 level recommended by Fornell and Larcker [92]. In sum (see table in S2 Table), these results indicate convergent validity of the measures.

To test for ***discriminant validity***, we calculated the difference between one model, which allowed the correlations between the constructs (with multiple indicators) to be constrained to unity (i.e. perfectly correlated), and another model, which allowed the correlations between the constructs to be free [93]. This was done for one pair of constructs at a time. For example, in testing CPROD and CMPERME, the chi-square difference test between the two models (χ^2^_d_(1) = 362.69, p < .001) affirmed the discriminant validity of these constructs. Similar results were obtained for the other chi-square difference tests, indicating discriminant validity.

To assess content marketing effectiveness, we drew on ***subjective measures***. A part of the literature on performance measurement tends to conclude that subjective measures, compared with objective measures, are less appropriate for performance assessments. It has been argued that managers may tend to overrate their organization’s performance [e.g., 94], and that using subjective measures can be problematic when explanatory variables of performance are measured using the same informant, as this can implicate common method bias [95]. However, as done in prior research [96], we deliberately decided to rely on managers’ subjective evaluations because of the lack of generally accepted and comparable objective content marketing effectiveness indicators. Moreover, Singh et al. [96] have demonstrated that carefully collected subjective performance measures can yield reliable and valid data. To alleviate ***common method concerns*** we first used *procedural remedies* in line with recommendations provided by Podsakoff et al. [95]. We divided the questionnaire into various subsections, so respondents were required to pause and carefully read instructions for each set of questions, contributing to the psychological separation of predictor and criterion measures. We relied on different scale types to reduce common scale properties. In addition, we kept items specific and labeled every point on the response scales to minimize item ambiguity. We also guaranteed anonymity to diminish the tendency to respond in a socially desirable manner, and we kept the questionnaire as short as possible to maintain motivation to respond accurately. In addition to these procedural remedies, we used the *regression-based marker variable technique* proposed by Siemsen et al. [97] to statistically control for potential method bias. According to this approach, common method bias can be effectively reduced when estimating a regression equation by adding a marker variable that is largely uncorrelated with the substantive variables of interest and suffers from some type of method bias. Hence, we deliberately included *impression management*, i.e. the conscious attempt to present oneself positively, as a potentially ideal marker variable into our study, based on the expectation that this measure is theoretically unrelated and similarly vulnerable to common method variance relative to other study variables. We measured the impression management form of social desirability via the three-item scale described by Winkler et al. [98]. Items were on 5-point agreement scales (1 = strongly disagree and 5 = strongly agree). Analysis of our data exhibited no to small bivariate correlations (< .15) of the impression management marker (*IMM*) with the substantive variables of interest, supporting the assumed unrelatedness. Thus, we added the marker variable to our regression analysis, described in more detail below, to control for potential common method bias.

#### Analysis

The study variables were on different response scales. Hence, we followed the recommendation from Cohen et al. [99] to put research findings into common, easily understandable metrics, and used simple linear transformations of the original scale units to convert the scores of all variables into standardized units of 0 to 100 (0, 100 for dichotomous variables), representing the percent of maximum possible (POMP) scores for each scale. This approach simplifies interpretability for example by giving immediate meaning to summary statistics such as means and measures of variability or by facilitating comparisons of scores across constructs.

We used linear multiple regression analysis for hypotheses testing in which all variables entered the regression equation on the same step. With regard to Hypothesis 3a, which predicts that content marketing is more effective when an intermediate number of media platforms is used, we categorized, as described above in the measures section, the originally continuous predictor variable so that an intermediate number of media platforms used was assigned the maximum value. Though such categorization is accompanied by loss of information, this allowed us to investigate whether CM effectiveness at an intermediate number of platforms used was different from when more or less platforms were used without resorting to a quadratic function. We proceeded analogously with regard to the analysis of Hypothesis 3b. Statistical analyses were performed using SPSS Statistics 24.0.0.1 software, reporting adheres to the SAMPL guidelines [100]. Prior to the main analysis, the assumptions of regression analysis were tested. To check linearity between the dependent and the independent variables, we employed partial residual plots of independent variables [101]. The plots exhibited only minor deviations from linear relations. Hence, we concluded that there was no major problem with the linearity assumption. Regarding multicollinearity, the highest value of variance-inflation factor was 2.81, and the highest value of the condition index equaled 24.90. Since these values are below the recommended threshold of respectively 10 and 30 [72], there is no indication for collinearity concerns. A Shapiro-Wilk test of the residuals (W(263) = 0.985, p < .01) found some evidence of nonnormality and a Koenker test (K = 29.97, p < .01) indicated presence of heteroscedasticity in the residuals. We therefore used the generalized information matrix (GIM) test described by King and Roberts [102] to detect potential model misspecification. Since the value (GIM = 1.375) is below the recommended threshold of 1.5, denoting that robust standard errors are not 1.5 times larger than classic standard errors, there is no indication for misspecification. Hence, we proceeded with our model, and to account for nonnormality and heteroscedasticity, we followed the recommendation of Dudgeon [103] to use HC3 as robust standard error estimator in our regression. Multiple regression with robust standard errors was carried out using the SPSS macro by Daryanto [104]. A p-value of < .05 was considered significant.

## Results

### Descriptive statistics

Table 1 lists the means, standard deviations, correlations, and Cronbach’s alphas of the study variables. In line with expectations, CMEFFECT related positively to CMSTRAT (r = .66, p < .001), to CPROD (r = .68, p < .001), to CMPERME (r = .61, p < .001), and to CMORG (r = .62, p < .001). Notably, CMEFFECT was not correlated with CDIST1, CDIST2, and CPROM.

**Table 1:** Means, standard deviations, correlations and Cronbach’s alphas of study variables.

Variables	M	SD	Items	1	2	3	4	5	6	7	8	9	10	11	12	13
1. CMSTRAT	71.58	19.65	4	.*86*												
2. CPROD	76.58	16.48	3	.60	.*71*											
3. CDIST1	45.75	28.91	1	-.20	-.01	--										
4. CDIST2	62.08	26.73	1	-.02	-.04	-.02	--									
5. CPROM	19.62	14.60	1	-.01	-.08	.02	-.06	--								
6. CMPERME	74.71	19.93	3	.63	.55	-.15	-.01	-.06	.*78*							
7. CMORG	68.35	21.05	4	.71	.57	-.30	.08	-.11	.66	.*84*						
8. SIZE 500–999^a^	22.05	41.54	1	.08	-.02	.02	.07	.06	-.03	.02	--					
9. SIZE 1,000–4,999 ^a^	27.38	44.67	1	.06	.08	-.04	-.01	-.05	.06	.08	-.33	--				
10. SIZE 5,000+ ^a^	24.33	42.99	1	.04	.07	.11	-.01	-.08	.14	.01	-.30	-.35	--			
11. SECTOR ^a^	74.52	43.66	1	-.06	-.03	-.01	-.14	.05	.02	.06	-.07	-.05	.13	--		
12. IMM	54.12	19.34	3	.12	.07	-.09	-.01	.03	.10	.13	.06	.04	-.06	.10	.*74*	
13. CMEFFECT	74.74	16.62	7	.66	.68	-.10	-.03	-.04	.61	.62	.03	-.03	.11	-.05	.13	.*88*

Notes: N = 263. POMP scores for all variables.

^a^ Dummy coded. All |r| > .11 are significant at p < .05, all |r| > .19, p < .01. Cronbach’s alphas for multi-item measures are in italics on the diagonal in the correlation matrix.

### Hypothesis testing

Results of the multiple regression analysis with CMEFFECT as dependent variable are presented in Table 2. The study variables explained a substantial proportion of variance in content marketing effectiveness (R^2^ = .61, F(12, 250) = 36.71, p < .001). In Hypothesis 1, we expected that there would be a positive association between a strong content marketing strategizing context, characterized by strategic clarity and strategy commitment, and content marketing effectiveness. The regression coefficient indicates that as we hypothesized, CMSTRAT is significantly and positively associated with CMEFFECT (β = .23, t(250) = 2.94, p < .01). Therefore, the data support Hypothesis 1.

**Table 2:** Determinants of content marketing effectiveness.

	CMEFFECT				
	B	SE(HC3)	β	t	p
Independent variables					
CMSTRAT	.193	.066	.228	2.94	.004
CPROD	.376	.075	.373	5.05	.000
CDIST1	.007	.024	.012	.29	.772
CDIST2	-.014	.028	-.023	-.50	.620
CPROM	.017	.042	.015	.41	.685
CMPERME	.148	.055	.178	2.69	.008
CMORG	.106	.054	.135	1.97	.049
Control variables					
SIZE 500–999	-.006	.019	-.015	-.30	.764
SIZE 1,000–4,999	-.036	.021	-.096	-1.73	.085
SIZE 5,000+	.007	.021	.018	.33	.743
SECTOR	-.020	.018	-.052	-1.12	.263
IMM	.045	.031	.052	1.42	.157
Model Statistics					
R^2^	.610				
Adjusted R^2^	.591				
F	36.712				
p value	< .001				

Note: N = 263.

With regard to Hypothesis 2, we predicted that a strong content production context, characterized by efforts to optimize customer-perceived content value and to adhere to normative quality criteria, should be associated with higher content marketing effectiveness. Results showed that CPROD was positively related to CMEFFECT (β = .37, t(250) = 5.05, p < .001). Thus, Hypothesis 2 cannot be rejected. Hypotheses 3a and 3b predicted that two aspects of content distribution, the usage of an intermediate number of media platforms and a joint deployment of print and digital media platforms, each affect content marketing effectiveness. However, results showed that CDIST1 (β = .01, t(250) = .29, p = .77) and CDIST2 (β = -.02, t(250) = -.50, p = .62) were not significantly related to CMEFFECT. Therefore, Hypotheses 3a and 3b are not supported by our data. Related to Hypothesis 3a, we conducted two exploratory post-hoc analyses to examine whether there might be (a) a linear relationship between the number of content distribution platforms used and content marketing effectiveness, or (b) an inverted U‐shaped relationship between the number of content distribution platforms used and content marketing effectiveness. With regard to (b), we introduced the square of the number of media platforms used as a new variable in the regression model in addition to the number of platforms used. With respect to Hypothesis 3b, we also conducted (a) a post-hoc analysis to test an alternative model that included the potential effect of focusing on print or digital media platforms on content marketing effectiveness, and (b) an analysis testing for a U‐shaped relationship between the share of content distribution budget allocated to digital media platforms and content marketing effectiveness. With regard to (b), we introduced the square of the budget share as a new variable in the regression model in addition to the budget share. However, none of these post-hoc analyses yielded significant effects. In Hypothesis 4, we predicted that there would be a positive relation between a strong content promotion context in terms of paid content promotion budgets and content marketing effectiveness. With respect to this hypothesis, CPROM was not found to have a significant impact on CMEFFECT (β = .02, t(250) = .41, p = .69). Hence, we find no support for Hypothesis 4. To further evaluate the relationship between content promotion and content marketing effectiveness, we conducted an additional exploratory post-hoc analysis. We tested an alternative model that assessed whether the number of content promotion measures is positively related to content marketing effectiveness. The number of measures was also not linked to content marketing effectiveness. Hypothesis 5 stated that content marketing is more effective when organizations have a stronger content marketing performance measurement context. Regarding this Hypothesis, the regression coefficient indicates that CMPERME is significantly and positively associated with CMEFFECT (β = .18, t(250) = 2.69, p < .01). This is the hypothesized outcome, and therefore the data support Hypothesis 5. Furthermore, a specialized content marketing organization with supporting processes and information technology systems (CMORG) was found to have a positive effect on content marketing effectiveness (CMEFFECT) (β = .14, t(250) = 1.97, p < .05), as we hypothesized in Hypothesis 6. Consequently, Hypotheses 6 cannot be rejected.

Finally, we conducted a robustness check of our results by adding the respective organization’s annual content marketing budget to the model. Including this variable into our model did not change our findings, all the variables that were significant remained significant, while the overall annual budget was not significant (β = -.04, t(245) = -0.70, p = .48).

## Discussion

This study examined whether and how the organizational context in which content marketing activities are embedded in determines content marketing effectiveness. We conceptualized and empirically tested a model that proposed that strong content marketing strategizing, content production, content distribution, content promotion, content marketing performance measurement, and structural and processual contexts drive content marketing effectiveness.

### Summary of findings and theoretical implications

Considered together, our analysis of the data reveals that context features have a substantial impact on the effectiveness of content marketing activities. Table 3 summarizes the findings.

**Table 3:** Summary of hypothesized results.

	CMEFFECT	
Content marketing context factors	Hypothesis	Supported
CMSTRAT	+	Yes
CPROD	+	Yes
CDIST1	+	No
CDIST2	+	No
CPROM	+	No
CMPERME	+	Yes
CMORG	+	Yes

Notes: + = a positive hypothesized relationship. Yes = the hypothesis was supported. No = the hypothesis was not supported.

Regarding the strategizing context, we found that a well-defined content marketing strategy that is clearly communicated, thoroughly understood by managers and employees, and widely supported within the organization positively influences content marketing effectiveness. The demonstration of this link between strategic clarity and strategy commitment on the one hand and content marketing effectiveness on the other hand adds to the theoretical and empirical elaboration of the determinants of content marketing effectiveness while incorporating insights from strategy research [24, 25, 27, 28] into the content marketing domain.

In addition, we found that a strong content production context, characterized by the optimization of customer-perceived content value and adherence to normative content quality criteria, has a significant, positive impact on content marketing effectiveness. Our results support the line of reasoning in the uses-and-gratifications- as well as information quality literature [29–32], that providing content aligned with a target group’s subjective judgement of usefulness will increase the likelihood that content is interacted with, in turn positively influencing content marketing effectiveness. While prior content marketing research focused on this argument [e.g., 3], we also introduce the compliance with normative content quality criteria (such as diversity of viewpoints or impartiality) as a novel content production context factor that positively influences content marketing effectiveness. From this perspective, the integration of research on journalistic quality in theories about content marketing effectiveness is essential for the progress of knowledge about content marketing effectiveness.

With regard to the content distribution context, we did not find that the usage of an intermediate number of media platforms has a positive influence on content marketing effectiveness. This finding is noteworthy since research on integrated marketing communications generally assumes that using multiple media platforms will increase the effectiveness of communications efforts but that deploying too many media properties will attenuate effectiveness [37, 38, 40, 41]. One reason for our result could be that the assumption of reactance theory underlying our hypothesis, that, from a certain point, the negative consequences of using an increasing number of media platforms outweigh the positive effects [41], does not hold. This explanation would be supported by a positive linear association between the number of content distribution platforms used and content marketing effectiveness. However, our post hoc analysis did not provide any evidence for this kind of relationship. Contrary to expectations, we also did not find a positive influence of a joint deployment of print and digital media platforms on content marketing effectiveness. In addition, post hoc analyses showed no significant effects of focusing on print or digital platforms only on CM effectiveness. These findings suggest that there is no general difference in effectiveness between these two kinds of media platforms, a result similar to the conclusion by Kwon and colleagues [105]. Heterogeneity of preferences theory suggests one interpretation for this [41], positing that media platform preference is idiosyncratic and that heterogeneity in individual platform preferences influences customer response to content marketing activities. Taking the aforementioned results together, the present study advances research on content marketing effectiveness by suggesting that effectiveness may be less a question of how many or whether print or digital content distribution vehicles are used, but more of utilizing precisely those media platforms that are best aligned with the respective organization’s target groups’ preferences. Following up on this, further research on the effects of using various content distribution platforms on content marketing effectiveness is warranted.

The present study did not find a positive relationship between paid content promotion budgets and content marketing effectiveness. This is not what we expected. However, empirical evidence from the field of advertising effectiveness research suggests an interpretation of the finding that more paid media investments are not always consistent with higher performance. According to respective descriptive knowledge [106], a metric that determines the level of performance is excess share of voice, defined as a brand’s share of voice minus share of market. Arguably, then, the amount invested in paid content promotion by a brand would have to be related to the total amount invested in paid content promotion in the brand’s category, and to the brand’s market position. Also, the contribution of paid content promotion to content marketing effectiveness could be shaped by the balance between paid promotion and owned content distribution platforms (e.g., [107]). This research therefore highlights that further work is needed to untangle the conditions under which paid content promotion measures might positively influence content marketing effectiveness.

Our theoretical elaboration and empirical investigation also provided evidence that core elements of the content marketing performance measurement context–regularly measuring the performance of print and digital content platforms and actually using the data obtained as guidance for continuously improving content offerings–positively influence content marketing effectiveness. Though previous research has shown positive performance implications of performance measurement in contexts other than content marketing [e.g., 55–57], this is the first study to successfully demonstrate this relationship for the content marketing domain. Our research thus expands previous research on CM effectiveness by incorporating performance measurement as a central element of a model of content marketing effectiveness. This finding might also have implications for future research, e.g. regarding the optimal configuration of content marketing performance measurement systems.

Finally, our work extends previous research on content marketing effectiveness by including structural specialization and specialization enabling processes and information technology systems as a new factor that positively influences content marketing effectiveness. The demonstration of the link between organizational structural and processual design elements on the one hand and content marketing effectiveness on the other hand lends support to researchers, such as Lee et al. [62], who have called for a new perspective of structural marketing, recognizing the importance of using organizational design elements to achieve marketing outcomes.

Overall, the aforementioned findings are important giving the centrality of empirical insights regarding the optimal design and implementation of content marketing initiatives to current academic interest [3, 5, 8].

### Management implications

The present study has important implications for practice as well. It clearly identifies four context factors that positively influence content marketing effectiveness. However, it is noteworthy that the strength of relationship between each of these factors and content marketing effectiveness varies. This implies, that managers could, e.g. if necessary due to budget or attention restrictions, prioritize improvements in the content marketing context factors in line with their order of importance for effectiveness as it was found in this study, being (1) content production context, (2) content marketing strategizing context, (3) content marketing performance measurement context and (4) content marketing organization. Nevertheless, efforts to drive improvement in a single context domain are less beneficial than a comprehensive effort to establish strong content marketing context conditions across the entire range of content marketing activities.

In the following sections, we present individual management recommendations, based on the order in which the various context areas in this study were found to be important.

We first advise managers to constitute a strong content production environment. To do so, we encourage content marketing executives to systematically evaluate and optimize customer-perceived content value, which means putting the audience and its needs and wants first while at the same time keeping an eye on the organization’s communications objectives without becoming self-centered. Moreover, our findings provide a powerful argument that organizations should not compromise on the journalistic quality of their content, but instead strive for creating content pieces that stand out regarding journalistic aspects such as narrative perspective, originality, diversity of viewpoints, accuracy, comprehensibility, or compliance with ethical standards.

Our findings also suggest that a strong content marketing strategizing context is associated with higher content marketing effectiveness. In this respect, managers should work towards establishing strategic clarity. To do so, crafting a compelling content marketing purpose and vision, formulating clear content marketing goals and objectives, defining content creation principles and standards, clarifying key stories and main topics, developing customer personas, investing care about what the most appropriate content formats would be for the audiences being targeted, or planning content that is matched to customers’ buying processes would be beneficial for marketers. In addition, our findings suggest that practitioners should pursue strengthening commitment to the content marketing strategy within the organization. Possible measures to enhance comprehension and backing of the content marketing strategy include regularly communicating its core pillars, rigorously and openly addressing areas of concern, explaining strategic decisions, continuously training employees, or fostering strategic conversations (e.g., [108]).

Third, we highly recommend establishing a strong content marketing performance measurement context because that would quite certainly go along with a higher level of content marketing effectiveness. Establishment of a strong content marketing performance measurement context requires content marketers to shift part of their content marketing budgets from actual content marketing initiatives to measurement and analytic efforts. Doing so would be counterproductive if it did not enhance content marketing effectiveness. Our research supports exactly such a reallocation of resources, demonstrating that it can positively affect content marketing effectiveness.

Fourth, our investigation implies that shaping the structural and processual context of content marketing activities is a central task of managers since a specialized organizational context unfolds positive effects on content marketing effectiveness. One promising way to advance structural specialization is setting up organizational platforms offering shared and specialized working environments, often referred to as brand newsrooms or content factories. Such platforms could include various desks dedicated to specific topics, media, and target groups, teams devoted to strategy, project management, and further service areas such as graphics, video, or analytics, and an editorial board ensuring integration. To unleash agility, these structures should be supported by processes and underlying information technology solutions enabling interaction and collaboration between content marketing specialists as well as integration with further marketing functions and other relevant organizational entities.

Finally, our study questions the current high level of practitioner enthusiasm for focusing on digital content distribution platforms and multichannel communications. In the light of this study’s findings, it seems to be beneficial for organizations to utilize precisely those media platforms and systems that are best aligned with the respective organization’s target groups’ preferences. Caution is also advised regarding practitioner enthusiasm for paid content promotion measures. “Pay to play” measures such as influencer marketing, social media advertising or native ads in editorial environments have been presented as indispensable means to boost content marketing reach and thus improve content marketing effectiveness. However, we do not observe any simple and direct positive effect of content promotion budgets on content marketing effectiveness. As this is one of the first investigations to examine the impact of paid content promotion in the content marketing domain and given that the use and functionality of content promotion measures evolve continuously, our findings are preliminary. Scholars and practitioners need to further explore this emerging field.

### Limitations and research directions

As all empirical research, the present investigation has limitations that call for attention in interpreting its findings. First, the data was cross-sectional which prohibits unambiguously interpreting the findings as indicating causality. Still, based on the theoretical argumentation provided above, the directions of causality implied in this study are likely. Future research might try to replicate these relationships via longitudinal or experimental study designs. A second limitation is that, though the study included organizations from various sectors and across different size categories, the sample is rather homogeneous with respect to cultural factors, as all participating organizations were located in Germany, Switzerland or Austria. Hence and given the global nature of content marketing research, scholars could investigate the suggested relationships in other contexts in order to further generalize the current findings. Third, the measurement of content marketing effectiveness is a potential limitation of this investigation, since we relied on subjective ratings rather than objective data. Thus, researchers might validate our findings with objective content marketing performance data. The study builds upon the views of a single key informant in every organization. While the key informant approach is common, relying on multiple informants from each organization might provide an even more balanced view. Besides, as earlier mentioned, the lack of any evidence of effects of the content distribution and content promotion contexts on content marketing effectiveness could be due to the way we framed them in this study. Therefore, other conceptualizations are worth investigating, including considering interactions of these context factors, as each factor’s contribution to content marketing effectiveness might be contingent upon the other. Also, only a limited number of potential confounders could be taken into account in this study. We adjusted for potential effects of firm size and industry, controlled for social desirability, and conducted an additional robustness check of our results that included the respective organization’s annual content marketing budget. In future, researchers could map out the nomological network of the research field in more detail using causal graph analysis [81], and subsequently conduct studies including further control variables to rule out alternative explanations for the observed relationships. Beyond addressing limitations, this study offers a number of additional directions for prospective research. For example, given that a strong content marketing performance measurement context offers demonstrable benefits, scholars might consider whether certain findings from the general marketing performance measurement field [e.g., 55, 109] also apply to the content marketing domain. Research might, e.g., explicitly take into account whether content marketing performance measurement is comprehensive or selectively focused on particular dimensions, because larger organizations could benefit from more comprehensive and smaller organizations from more focused approaches. Furthermore, future studies may explore the influence of the organizational content marketing context on content marketing effectiveness via structural characteristics other than specialization. Other major structural characteristics, such as centralization, formalization, or modularity, might also exert influence on content marketing effectiveness. Importantly, future research might investigate mediating or moderating variables, such as external environmental effects. Market turbulence, for example, may moderate the value of content marketing context factors. Such investigations could further deepen the understanding of the determinants of content marketing effectiveness.

## Supporting information

S1 Table Measurement of main variables. (DOCX)

S2 Table Factor loadings, composite reliability estimates, average variance extracted. (DOCX)

S1 File Dataset of the study. (SAV)

S1 Appendix Literature review on CM effectiveness. (DOCX)
