# Supplementary material for: Determinants of content marketing effectiveness: Conceptual framework and empirical findings from a managerial perspective
Source: PLoS One. 2021 Apr 1;16(4):e0249457. doi: 10.1371/journal.pone.0249457 (PMC8016322; doi:10.1371/journal.pone.0249457)
Supplement: S1 Table — (DOCX) [file pone.0249457.s001.docx]

**S1 Table. Measurement of main variables.**

| **Construct** | **Items** | **Scale** |
| --- | --- | --- |
| CMSTRAT  based on [73, 74], CA^*^ 0.70- 0.98 | We have a well-developed content marketing strategy.  Our content marketing strategy is clear and understandable.  Our content marketing strategy is long-run oriented.  Our content marketing strategy is strongly supported by managers and employees. | Four-item, five-point Likert type scale, anchored by ‘strongly disagree’ and ‘strongly agree’ |
| CPROD  based on [3, 35, 75], CA^*^ 0.84-0.87 | The content needs of our target groups have priority in content creation.  We base our content production on journalistic principles (e.g. comprehensibility, timeliness, accuracy).  We plan and create content systematically. | Three-item, five-point Likert type scale, anchored by ‘strongly disagree’ and ‘strongly agree’ |
| CDIST1  based on [76] | Please mark the media platforms used by your organization for content distribution purposes.  customer magazines or newspapers  corporate books  company reports  owned digital media (websites, apps, newsletters, blogs)  organic social media  paid social media  emerging platforms (e.g. chatbots, voice assistants) | Multiple-selection list with seven items |
| CDIST2  based on [77] | How is your organization’s annual content distribution budget divided between digital media and print media?  Please state the shares that are allocated to digital and print media, respectively (in total 100%). | Two linked sliders, each ranging from 0% to 100%, total limited to 100% |
| CPROM  based on [77] | How is your organization’s annual content marketing budget divided between different activities?  Please state the share that is allocated to content promotion measures. | Slider, ranging from 0% to 100% |
| CMPERME  based on [55, 56], CA^*^ 0.72 | We frequently measure the performance of the digital media employed in our content marketing initiatives.  We frequently measure the performance of the print media employed in our content marketing initiatives.  We use performance measurement data for continuously improving our content offerings. | Three-item, five-point Likert type scale, anchored by ‘strongly disagree’ and ‘strongly agree’ |
| CMORG  based on [63, 66, 78, 79], CA^*^ 0.77 | Our organization has a dedicated content marketing unit.  Our organization has a large number of “specialist” employees who direct their efforts to content marketing activities.  The organizational structure and processes support smooth execution of content marketing activities.  Our organization makes use of information technology systems that support content marketing activities (content marketing platforms). | Four-item, five-point Likert type scale, anchored by ‘strongly disagree’ and ‘strongly agree’ |
| CMEFFECT  based on [69-71], CA^*^ 0.75- 0.84 | How effective is your organization's content marketing with regard to the following aspects?  Gets our target groups to think about our brand.  Triggers positive emotions among our target groups.  Activates our target groups to delve into our brand.  Convinces our target groups that the promise made by our brand can be relied upon.  Strengthens the confidence that we act in our target groups’ best interest.  Contributes to evaluating our brand favorably.  Increases the value of our brand. | Seven-item, five-point Likert type scale, anchored by ‘strongly disagree’ and ‘strongly agree’ |

Note: ^*^ CA refers to the Cronbach’s alpha scores in the original studies where available.
