# Supplementary material for: Determinants of content marketing effectiveness: Conceptual framework and empirical findings from a managerial perspective
Source: PLoS One. 2021 Apr 1;16(4):e0249457. doi: 10.1371/journal.pone.0249457 (PMC8016322; doi:10.1371/journal.pone.0249457)
Supplement: S2 Table — (DOCX) [file pone.0249457.s002.docx]

**S2 Table. Factor loadings, composite reliability estimates, average variance extracted.**

| **Construct** | **Standardized factor loadings** | **Composite reliability** | **Variance extracted** |
| --- | --- | --- | --- |
| CMSTRAT  (4 Items) | .68-.85 | .86 | .62 |
| CPROD  (3 Items) | .58-.80 | .71 | .45 |
| CDIST1  (1 Item) | .92 | .85 | .85 |
| CDIST2  (1 Item) | .92 | .85 | .85 |
| CPROM  (1 Item) | .92 | .85 | .85 |
| CMPERME  (3 Items) | .72-.79 | .78 | .55 |
| CMORG  (4 Items) | .72-.81 | .84 | ,56 |

Note: N = 263.
