# Supplementary material for: Determinants of content marketing effectiveness: Conceptual framework and empirical findings from a managerial perspective
Source: PLoS One. 2021 Apr 1;16(4):e0249457. doi: 10.1371/journal.pone.0249457 (PMC8016322; doi:10.1371/journal.pone.0249457)
Supplement: S1 Appendix — (DOCX) [file pone.0249457.s004.docx]

S1 Appendix. Literature review on CM effectiveness.

Any empirical investigation of the determinants of content marketing effectiveness requires a proper conceptualization of CM effectiveness. In order to conceptualize CM effectiveness, we performed a literature review in April 2020 searching EBSCO Business Source Complete, EBSCO Communication & Mass Media Complete and Web of Science. We searched titles and abstracts of the databases for combinations of the global keyword “content marketing” with “effectiveness”, “efficacy”, “effective”, “effect”, “performance”, “impact” or “consequence”. The search was automatically limited by considering only articles in peer reviewed journals in English. The hits were pooled, and doubles were manually eliminated. A total of 77 articles resulted from the search.

Referring to these publications, it is evident that there is still a level of debate in the literature surrounding the definition of content marketing, and thus it comes to no surprise that this also holds for the understanding of CM effectiveness. However, referring to the identified literature, CM effectiveness in the broadest sense denotes the degree to which content marketing helps reaching the focal organization’s strategic business objectives [see, e.g. 1-3]. In addition to this basic consent, there are three more aspects that are commonly mentioned when it comes to CM effectiveness with respect to consumers or business customers as target groups. First, taking Wang et al. [2] as an example, the literature stresses that CM effectiveness relates to the level of customer engagement realized at the appropriate points in their buying decision processes. Second, CM effectiveness materializes in the degree of established positive brand attitudes of and trusted relationships with customers [4]. Third, CM effectiveness is finally reflected in positive business outcomes in terms of prospects’ or customers’ favorable responses to a brand and its communications (see e.g. [2]).

In a similar vein, Hollebeek and Macky [1] proposed a framework that differentiates three tiers of content marketing consequences. For them, first-tier CM consequences include consumers' cognitive and emotional engagement, denoting brand-related thought and mental elaboration, and brand-related affect, respectively [5]. Over multiple interactions, these types of engagement might trigger brand-related sense-making [6] and identification [7]. Furthermore, first-tier CM consequences also comprise behavioral engagement, i.e. a target group’s energy, effort and time spent on a brand [3], which can foster consumer citizenship behavior [8]. As a result, and as second-tier consequences, content marketing might affect consumers’ brand trust in terms of credibility in the brand and confidence in its motives, and consumers’ brand attitudes expressed by more favorable brand evaluations [9, 10]. Improved brand trust and attitudes could in turn lead to a higher level of brand equity [11], implying an increased consumer's perceived value level of a brand, as third-tier consequences.

In line with these considerations, content marketing activities can be seen as effective if they trigger superior levels of cognitive, emotional and behavioral customer engagement at the appropriate points throughout the customer journey, strengthen customers’ brand trust and induce favorable brand attitudes, and increase customers’ perceived value of a brand, leading to more favorable responses to the brand and its communications, and thus helping the focal organization reach its strategic business objectives.

References

1. Hollebeek LD, Macky K. Digital Content Marketing's Role in Fostering Consumer Engagement, Trust, and Value: Framework, Fundamental Propositions, and Implications. Journal of Interactive Marketing 2019 Feb; 45: 27-41. <https://doi.org/10.1016/j.intmar.2018.07.003>.
2. Wang WL, Malthouse EC, Calder B, Uzunoglu E. B2B content marketing for professional services: In-person versus digital contacts. Industrial Marketing Management 2019 Aug; 81: 160-168. <https://doi.org/10.1016/j.indmarman.2017.11.006>.
3. Holliman G, Rowley J. Business to Business Digital Content Marketing: Marketers' Perceptions of Best Practice. Journal of Research in Interactive Marketing 2014; 8(4): 269-293. <https://doi.org/10.1108/JRIM-02-2014-0013>.
4. Vollero A, Palazzo M. Conceptualizing content marketing: a delphi approach. Mercati & Competitività 2015; 1: 25-44; <http://dx.doi.org/10.3280/MC2015-001003>.
5. Harrigan P, Evers U, Miles M, Daly T. Customer Engagement and the Relationship Between Involvement, Engagement, Selfbrand Connection and Brand Usage Intent. Journal of Business Research 2018 Jul; 88: 388-396. <https://doi.org/10.1016/j.jbusres.2017.11.046>.
6. Basu K, Palazzo G. Corporate Social Responsibility: A Process Model of Sensemaking. Academy of Management Review 2008; 33(1): 122-136. <https://www.jstor.org/stable/20159379>.
7. Tuskey U, Golob U, Podnar K. The Role of Consumer-Brand Identification in Building Brand Relationships. Journal of Business Research 2013; 66(1): 53-59. <https://doi.org/10.1016/j.jbusres.2011.07.022>.
8. Gruen T. The Outcome Set of Relationship Marketing in Consumer Markets. International Business Review 1995; 4(4), 447-469. <https://doi.org/10.1016/0969-5931(95)00026-7>.
9. Ganesan S, Hess R. Dimensions and Levels of Trust: Implications for Commitment to a Relationship. Marketing Letters 1997; 8(4): 439-448. <https://www.jstor.org/stable/40216470>.
10. Park CW, MacInnis D, Priester J, Eisingerich A, Iacobucci D. Brand Attachment and Brand Attitude Strength: Conceptual and Empirical Differentiation of Two Critical Brand Equity Drivers. Journal of Marketing 2010; 74(6): 1-17. <https://doi.org/10.1509/jmkg.74.6.1>.
11. Keller KL. Conceptualizing, Measuring, and Managing Customer-Based Brand Equity. Journal of Marketing 1993; 57(1): 1-22. <https://www.jstor.org/stable/1252054>.
